# Biomechanical considerations in RPD design: application and perspective of finite element method in distal extension removable partial denture rehabilitation

**DOI:** 10.3389/fdmed.2025.1667504

**Published:** 2025-10-03

**Authors:** Yixuan Zhu, Jiangqi Hu, Bin Luo, Yafei Yuan, Qingsong Jiang

**Affiliations:** ^1^ School of Stomatology, Capital Medical University, Beijing, China; ^2^ Department of Prosthodontics, Beijing Stomatological Hospital, Capital Medical University, Beijing, China

**Keywords:** removable partial denture (RPD), finite element analysis (FEA), biomechanics, digital dentistry, artificial intelligence (AI)

## Abstract

Removable partial dentures (RPDs) remain a widely used and cost-effective solution for patients with dentition defects. However, their long-term success, particularly in distal extension cases, depends heavily on biomechanical performance. Finite element analysis (FEA) has emerged as a valuable tool for evaluating stress distribution and guiding RPD design. This review synthesizes FEA-based insights into key biomechanical parameters—including abutment selection, clasp geometry, rest position, major connector stiffness, and material properties—with a particular focus on Kennedy Class I and II scenarios, and special attention to implant-supported RPDs (ISRPDs). Recent developments in digital workflows, such as intraoral scanning and CAD/CAM fabrication, have further enabled personalized modeling and rapid optimization. In addition, the integration of artificial intelligence (AI) with FEA shows promises in automating framework generation, predicting stress outcomes, and supporting closed-loop design optimization. While these technologies offer exciting potential, current models still lack integration of patient-specific factors such as mucosal properties, saliva, and gag reflex, contributing to discrepancies between simulations and clinical outcomes. Bridging this gap through improved modeling and data-driven approaches will be key to delivering personalized, biomechanically optimized RPD solutions.

## 1 Introduction

With rising life expectancy and an aging population, the prevalence of partial edentulism among adults is on the rise (1). A retrospective epidemiological analysis from 1995 to 2015 highlighted that dentition defect rate was prevalent among 86.1% of Chinese adults aged 65–74 years (2). This demographic exhibited a significant unmet need for prosthodontic rehabilitation, with both the incidence of tooth loss and the corresponding demand for restorative interventions progressively increasing with advancing age.

**Figure 1:**
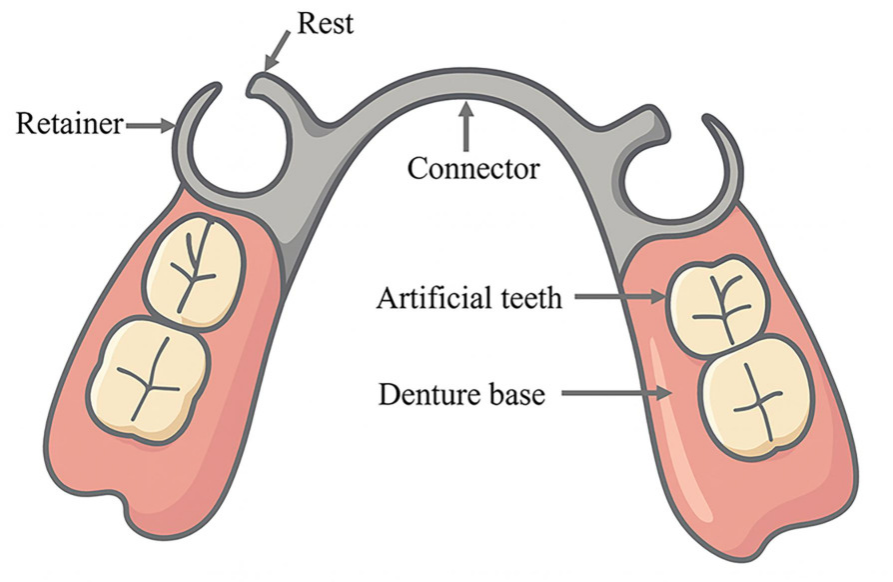
Schematic diagram of major components of an RPD, including the major connector, clasp, rest, and denture base. The illustration was created by the authors using Adobe Illustrator.

Removable partial dentures (RPDs), as the most employed approach for restoring partially edentulous dentitions, consist of several key components—including artificial teeth, denture base, rests, retainers, and connectors (Figure 1)—which work together to provide retention, support, and stability. They offer advantages such as varied indications, cost-effectiveness, and ease of repair. However, the potential biomechanical risks associated with RPDs—including abutment tooth loosening, cantilever effects at distal extension sites, and pressure-induced alveolar bone resorption—should not be overlooked, particularly in patients with distal extension tooth loss (3).

**Figure 2:**
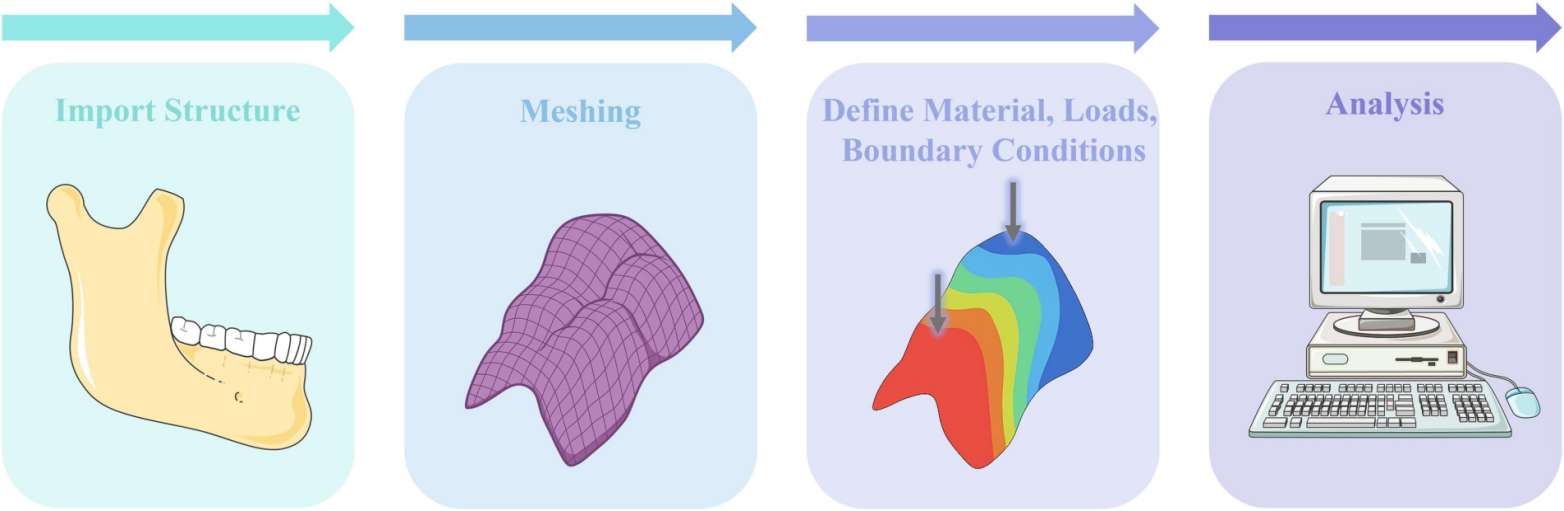
Illustrated workflow of FEA for RPD biomechanics: (1) import anatomical structures, (2) meshing, (3) define material properties, loads, and boundary conditions, and (4) perform stress/strain analysis. The illustration was created by the authors using BioRender.

Biomechanics is a branch of biophysics that applies the principles and methods of mechanics to quantitatively study mechanical phenomena in biological systems. Its scope spans from the entire organism to systems and organs—including blood, bodily fluids, internal organs, and skeletal structures (4). The foundational laws of biomechanics are the conservation of energy, conservation of mass, and the laws of momentum, complemented by constitutive equations (5). The biomechanics of RPD rehabilitation plays a crucial role in the long-term success of prosthodontic treatment (6). However, current RPD designs lack universally accepted, systematic biomechanical guidelines, resulting in substantial variability in RPD design decisions among different practitioners, even for identical clinical scenarios. This variability significantly impacts the long-term outcomes of RPD treatments and patient satisfaction. Finite element analysis (FEA) offers a powerful computational approach in biomechanics by enabling precise simulation of stress, strain, and deformation in complex biological structures under various loading conditions, which are limited in clinical trials due to ethical and practical constraints (7). The typical workflow of FEA includes importing structure, meshing, assignment of material properties, application of boundary conditions and loads, analysis and post-processing of results (8) (Figure 2).

In biomechanical studies of RPDs, finite element models commonly apply zero displacement boundary conditions on the outer surface of the abutment tooth roots to simulate the intrinsic support provided by the periodontal ligament and alveolar bone (9). The free-end mucosa exhibits viscoelastic compliance. To capture this behavior, Ramakrishnan et al. (10) introduced an adhesive viscoelastic layer between the RPD and mucosa, modeled using the Prony series approximation to simulate soft tissue compliance effectively. In terms of material properties, frameworks such as cobalt–chromium alloys, with a Young's modulus of approximately 218,000 MPa and a Poisson's ratio of about 0.3 are usually defined as isotropic, linearly elastic materials (11). The periodontal ligament is a non-homogeneous and anisotropic tissue that does not exhibit linear elastic behavior (12). However, this characteristic is often neglected in dental FEAs. In most studies, the periodontal ligament is simplified as a linearly elastic, homogeneous, and isotropic material model to reduce modeling complexity and computational demand, which leads to discrepancies between the simulation outcomes and the actual biomechanical environment of the oral cavity (13).

This paper aims to discuss RPD designs that minimize potential biomechanical risks by reviewing FEA-based biomechanical distributions within natural (abutments, alveolar bone, mucosa) and artificial (implants) supporting structures, as well as their interactions with prostheses. We not only synthesize existing FEA-based biomechanical evidence for distal extension RPDs (Kennedy Class I and II) but also bridge these insights with emerging digital workflows and AI-assisted modeling, highlighting how FEA can evolve from a theoretical simulation tool into a practical, patient-specific design strategy.

## 2 Biomechanical analysis under functional conditions of RPDs

**Figure 3:**
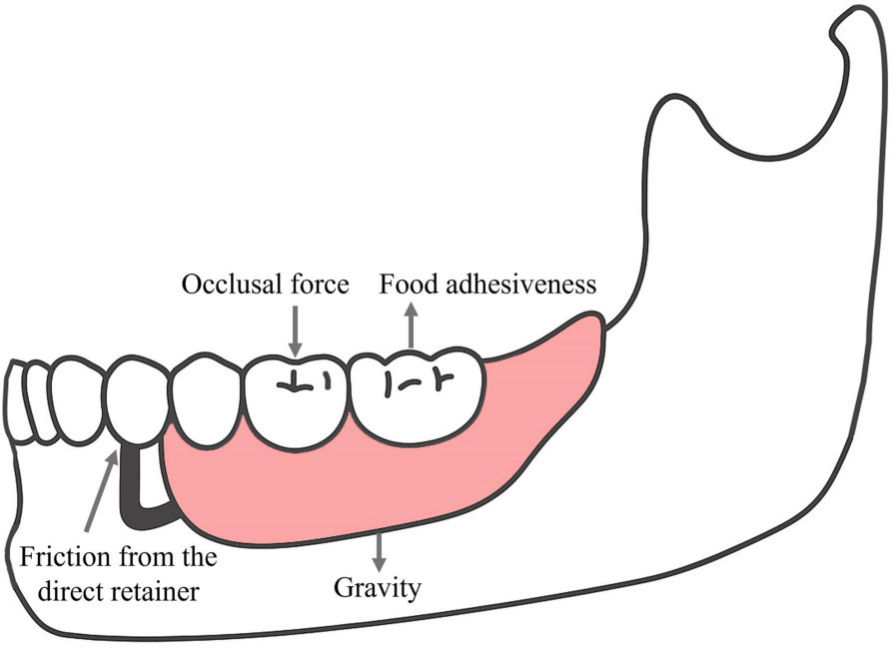
Force analysis of a removable partial denture (RPD) under functional conditions. The diagram was created with Adobe Illustrator by the authors. Multi-directional forces acting on an RPD include occlusal force, food adhesiveness, friction from the direct retainer, and gravity.

Under functional conditions, RPDs are subjected to several forces, including vertical dislodging forces caused by gravity (in maxillary RPDs) and food adhesiveness, as well as vertical and lateral forces generated by occlusal loading. The friction between the direct retainer and the abutment tooth constitutes the primary source of retention force resisting the dislodging forces on the RPD (3) (Figure 3). Distal extension edentulism (Kennedy Class I and II) is one of the most common types of partial edentulism, accounting for 19.51% among patients with dentition defect (14). In distal-extension RPD cases, a combination-type support design is commonly employed. The terminal abutment acts as a cantilever under occlusal loading, and the differential compliance between the abutment and the mucosal tissues results in asynchronous deformation. This mismatch can lead to stress imbalance across the rigid cast framework, producing excessive distal torque on the abutment adjacent to the edentulous area. Over time, such unfavorable loading may compromise the periodontal integrity of the abutment, potentially causing its mobility or even loss (15). FEA of distal-extension RPDs reveals that stress on the terminal abutment is concentrated in the apical and distal regions, promoting distal tipping, which may disrupt proximal contact with adjacent teeth, induce secondary occlusal trauma, and accelerate alveolar bone resorption (16).

In mandibular Kennedy Class II arches, Suenaga et al. (17) found that both pressure sensor measurements on the tissue surface of the denture base and FEA indicated a consistent stress distribution pattern, with stress predominantly concentrated on the lingual side of the distal extension ridge. During functional loading of RPDs, the components subjected to the highest stress include the lingual minor and major connectors of the terminal abutment, the clasp's horizontal curvature of the approach arm (18), and the junction between the clasp arm and body in Aker clasps (19). Moreover, the reciprocal arm experiences greater stress than the retentive arm (19).

## 3 FEA-guided design strategies for RPDs

### 3.1 Selection of abutment teeth

The optimal number of abutment teeth for RPDs is typically 2–4. The natural tooth adjacent to the edentulous space is preferred as the primary abutment (3). For Kennedy Class I and II dentures, the number and position of abutment teeth directly determine the configuration of the major connector, thereby influencing the rigidity and mobility of the framework. In addition to the abutment adjacent to the edentulous space, restoring a Kennedy Class II arch typically requires support from contralateral abutment. Without such cross-arch support, the denture and its abutment teeth are nearly incapable of resisting the bending forces exerted on the abutment (20). An FEA study (21), in which the abutment adjacent to the edentulous space in a Kennedy Class II arch was fixed, evaluated the effect of varying the position of the contralateral abutment (canine, first premolar, second premolar, or first molar). Quantitative comparisons of displacement and intrusion, together with the finite element model parameters, are summarized in Table 1. The results showed that selecting the contralateral canine as the second abutment significantly increased distal extension displacement under oblique loading. However, under vertical loading, both the displacement of the distal extension and the stress distribution within the mucosa were relatively insensitive to the position of the contralateral abutment.

**Table 1:** Material properties, boundary conditions, loading settings, and displacement outcomes of finite element models with different contralateral abutment selections. Data adapted from Ref (21).

Contralateral abutment	Canine	First premolar	Second premolar	First molar
Framework properties	Poisson's ratio of 0.3 and a modulus of elasticity of 200 GPa			
Mucosal properties	Poisson's ratio of 0.45 and a modulus of elasticity of 3.4 × 10^−3^ GA			
Boundary conditions	The alveolar mucosal surface and the rest adjacent to the edentulous ridge were fixed vertically; the rest on the contralateral abutment was constrained in all directions.			
Loading conditions	·60 N vertical loading (VL) ·60 N oblique loading (OL, 10° buccal direction)			
Framework displacement (VL, μm)	77	76	71	80
Framework displacement (OL, μm)	168	112	88	115
Vertical intrusion (VL, μm)	69	68	63	71
Vertical intrusion (OL, μm)	78	77	72	81

The unfavorable biomechanical performance of the canine under oblique loading can be attributed to the pronounced lingual inclination of the canine's anatomical structure. Placing a rest on such an inclined surface may generate an unfavorable lever effect on the abutment, making the denture more susceptible to sliding under occlusal loading (22). From a clinical perspective, the increased displacement and lever action associated with a canine abutment may not only accelerate periodontal breakdown of the abutment tooth and compromise its long-term prognosis (23), but also increase the risk of mucosal trauma, soreness, and denture base instability, thereby necessitating more frequent relining or adjustments and reducing patient compliance and satisfaction (24) In addition, placing a metal clasp on a canine abutment is often associated with complaint about esthetics (25). In contrast, selecting a more posterior contralateral abutment, such as the second premolar, provides a more favorable fulcrum to control rotational movement and distribute occlusal forces more evenly (21), which may contribute to improved denture stability, reduced soft tissue injury, preservation of periodontal health, and ultimately enhanced masticatory efficiency and patient-reported outcomes.

Nevertheless, it should be acknowledged that the findings of this study are derived from FEA simulations, which inevitably involve several simplifying assumptions. For instance, the actual mandibular kinematics is highly intricate and exhibits marked inter-individual variability (26). In addition, systemic diseases such as osteoporosis may reduce bone mineral density and thereby increase denture base displacement, while patient-specific anatomical differences may further lead to discrepancies between simulated and real-world outcomes (27). These factors limit the direct clinical transferability of FEA results. Therefore, future *in vitro* experiments and long-term clinical follow-up studies are warranted to validate the biomechanical predictions of FEA simulations and to establish evidence-based guidelines for abutment selection in RPD design.

### 3.2 Configuration and placement of occlusal rest

The occlusal rest is an essential component of conventional RPDs, which provides support, transmits occlusal forces, stabilizes the prosthesis, prevents food impaction, and helps restore proper occlusal relationships (3). Sato et al. (28) found that stress is primarily concentrated at the junction between the rest seat and the minor connector. Increasing the thickness and width of the rest significantly enhances its yield strength, whereas increasing its length is associated with reduced yield strength. And a right-angle junction between the rest and the minor connector can reduce stress concentration on the minor connector. Regarding the selection of occlusal rest position, although it was documented (29) that distal rests may lead to distal tipping of the abutment, increased tooth mobility, and alveolar bone resorption. Muraki et al. (30) revealed by FEA that both displacement and stress remained within the physiological limits of the tissues, regardless of whether the occlusal rest was placed on the mesial or distal side of the terminal abutment. The use of mesial occlusal rests was recommended by another study (21), as placing the rest on the mesial side of the abutment adjacent to the distal extension was shown to reduce stress and displacement in both the abutment and the underlying soft tissues under vertical loading, with forces more closely aligned with the long axis of the abutment tooth.

### 3.3 Clasp design

The direct retainers of conventional RPDs are primarily clasps. For circumferential clasps, stress is concentrated at the junction between the clasp arm and the body, whereas in bar clasps, the highest stress occurs at the junction between the clasp and the minor connector (31). The retentive forces of the clasps are influenced by the length of the clasp arm, the radius and shape of the cross-section, as well as the lengths of the vertical and horizontal beams (in I-bar clasps) (32). As the length of the I-bar clasp arm increases, the risk of failure due to fatigue or plastic deformation rises markedly. Studies have shown that when the horizontal arm (L_1_) extends to 7–9 mm or the vertical arm (L_2_) to 6–8 mm, stresses within the clasp can reach approximately 500 MPa, corresponding to the critical zone of CoCr alloy. When the length further exceeds 8–9 mm, stress levels may escalate to 500–900 MPa, thereby surpassing the yield strength and fracture limit of cobalt-chromium. Therefore, to minimize the risk of failure, the optimal design length of both L_1_ and L_2_ in I-bar clasps should not exceed 6 mm (32), which makes them more suitable for teeth with smaller mesiodistal dimensions, such as canines and premolars (33). In molar designs, L_1_ and L_2_ of an I-bar clasp often exceed 8–9 mm in length, requiring an increase in clasp cross-sectional radius to compensate for the adverse effects of longer L_1_ and L_2_. For example, when L_2_ is extended by 4 mm, the radius must be increased from 0.7 mm to 1.0 mm to maintain stress within a safe range; however, this nearly doubles the clasp volume and results in greater stress concentration transmitted to the abutment tooth (32). In the RPI system, stress tends to concentrate on the internal surface of the retentive arm and in the region directly overlying the vertical projection of the horizontal beam (34). Sato et al. (35) demonstrated by FEA that I-bar clasps with a curvature radius of about 3 mm, a taper of 0.020–0.023, and a thin-wide cross-section generated the lowest stresses, whereas deviating from these ranges increased von Mises stress by 30%–100%, raising the risk of clasp fatigue and enamel overload.

For circumferential clasps, the shorter the clasp arm, the greater the perpendicular force it exerts on the abutment during dislodgement, resulting in increased frictional retention. Clinically, the recommended length for an Aker clasp is 8–12 mm (36). In the model of Chen et al. (37), the retentive force of a bending clasp at 6 mm was nearly four times that at 10 mm, whereas at 12 mm it was reduced to about half of 10 mm. The shape and thickness of the clasp tip also have a significant impact on the mechanical properties of the clasp. Sandu et al. (34) found that a semicircular clasp with a diameter of 1.0 mm (max von Mises stress ≈ 1,201 MPa, displacement ≈ 0.41 mm) exhibited similar rigidity and deflectionto a circular clasp with a 0.7 mm diameter (max von Mises stress ≈ 1,381 MPa, displacement ≈ 0.56 mm). The semicircular design reduced stress concentration on the enamel and offered improved patient comfort. However, a round clasp is able to flex in all directions, while a half-round clasp is restricted to bidirectional flexure, which makes the former better dissipate harmful forces during functional movements of the denture (3).

Although biomechanical studies have repeatedly emphasized the stress concentration regions and differences in fatigue resistance among various clasp and framework materials, their clinical significance is equally noteworthy. Higher stress concentration at the clasp–tooth interface has been associated with an increased risk of abutment tooth mobility, periodontal breakdown, and even tooth loss. Muraki et al. (30) reported that even minimal vertical displacement of distal-extension clasps, although within the physiological tolerance, induced localized compressive stress in the periodontal ligament. Similarly, Rungsiyakull et al. (38) demonstrated in an FEA model that periodontal tissues around the abutment root surface and gingival tissues covering the edentulous ridge were prone to displacement and deformation under functional loading. Therefore, clasp designs and materials that reduce stresses on abutment teeth and supporting soft tissues may help minimize periodontal damage of the abutments and improve patient comfort and compliance (21, 39). At the same time, adequate fatigue resistance of the clasp should be ensured to lower the risk of fracture and thereby prevent the inconvenience and complaints associated with denture repair (40). Overall, these associations suggest that biomechanical optimization is not merely of laboratory relevance but should also translate into tangible benefits in preserving oral tissue health, extending abutment longevity, and enhancing patient satisfaction.

Due to differences in experimental methods, materials, and fabrication techniques, study findings remain inconsistent. To date, no biomechanically validated optimal clasp parameters have been established, underscoring the need for computational approaches to optimize stress distribution within clasp designs. Representative FEA studies evaluating different clasp materials, designs, and biomechanical outcomes are summarized in Table 2. Additionally, emerging materials such as PEEK, PEKK, and nanocomposites have shown potential in improving biomechanical behavior of clasp systems and are further discussed in Section 3.5.

**Table 2:** Studies reporting the influence of different clasp materials and designs on stress distribution, retention, and displacement in conventional RPDs.

Author (year)	Materials	Clasp type	Key findings	Quantitative comparison
Tribst et al. (2020) (41)	Polyamide; Polyoxymethylene; PEEK; Gold; Titanium; CoCr	Aker clasps	Rigid materials and deeper undercuts increased clasp stress and enamel risk but improved retention.	Polyamide (0.25 mm undercut) showed the lowest clasp stress (17.1 MPa), enamel stress (1.4 MPa), and retention force (3.13 N), whereas CoCr (0.75 mm undercut) showed the highest values (297.9 MPa, 46.4 MPa, 65.4 N).
Sandu et al. (2010) (34)	Stainless steel wrought wire	Retentive clasps with round &half-round cross-sections	Half-round 1.0 mm provided similar stiffness as 0.7 mm round but with lower enamel stress and better comfort.	Displacement under 5 N load: 0.7-mm round = 0.41 mm; 1.0-mm half-round = 0.56 mm; Maximum von Mises stress: 0.7-mm round = 1,201 MPa; 1.0-mm half-round = 1,381 MPa
Peng et al. (2019) (31)	PEEK; CoCr	PEEK clasps with various taper/thickness ratios	PEEK clasps generated lower abutment stresses than metal clasps, with adequate retention and superior esthetics,	Deformation after 15,000 cycles: CoCr = 0.017 mm; PEEK = 0.011–0.017 mm (*P* > 0.05); Load for deflection: CoCr = 8.26 ± 0.55 N; PEEK = 2.06 ± 0.09–3.67 ± 0.17 N (*P* < 0.05).
Richert et al. (2021)	CoCr	I-bar clasp	Optimal performance when vertical arm (L_1_) and horizontal arm (L_2_) ≤ 6 mm; stresses rise above yield limits if L_1_ > 8 mm or L_2_ > 9 mm. Increasing clasp radius mitigates stress concentration.	Analytical model: Stresses <400 MPa at L1, L2 ≤ 6 mm; 500–900 MPa at L1 = 8 mm, L2 = 9 mm. FEA: max stress 440 MPa at clasp root under 5 N load; deflection ≈0.238 mm, consistent with analytical model (0.25 mm).
Yamazaki et al. (2019)	Resin	Thermoplastic resin clasps with different block-out designs	Horizontal block-out reduced resin clasp retention; FEA showed peak stress under clasp shoulder, increasing with block-out width.	There was no statistically significant difference in retention between polyester and polyamide; retention depended primarily on the undercut rather than the resin material.
Zarrati et al. (2015) (42)	NiCr	I-bar with mesial or distal rests	Stress concentrated at cervicobuccal region, distal proximal plate, and middle third of root. Adding both mesial & distal rests reduced tooth movement.	Maximum von Mises stress distributions in all models were located in the I-bar placed in the buccocervical region of abutments (15–30 MPa).
Chen et al. (2024) (37)	CoCr	Rod-shaped and bending clasps	For rod models, retention decreased with the cube of length (L) and increased with the fourth power of diameter (D). For bending models, retention was inversely proportional to the cube of base width (W) and inversely proportional to the height (H).	Increasing L from 10 to 15 mm reduced retention by ∼70%; increasing D from 1.0 to 1.2 mm doubled retention. Stress peaked at clasp base in all cases.

### 3.4 Major connector design

The major connector plays a crucial role in distributing occlusal forces within the denture and must possess sufficient rigidity to transmit and evenly distribute these forces to the abutment teeth and adjacent supporting tissues. If the connector lacks adequate stiffness, it may flex under occlusal load, resulting in repeated compression of the mucosa and underlying bone, which may lead to mucosal inflammation and alveolar bone resorption (43). The stress generated in the major connector depends on its material, design type, shape, and thickness (44).

For maxillary RPDs, FEA has shown that the anteroposterior palatal strap exhibits greater rigidity compared to the full palatal plate, posterior palatal strap, and horseshoe-shaped palatal plate, with the latter demonstrating the least stiffness (45). The shape of the palate also influences the stress and displacement of the major connector: in narrow and deep palates, displacement is minimal, whereas wider and shallower palates are associated with greater movement (46).

To avoid damage caused by flexing of the major connector, cast alloys with low flexibility should be used to ensure adequate stiffness. Moreover, increasing the contact area between the connector and supporting tissues becomes especially important as connector length increases, significantly contributing to denture stability. In mandibular Kennedy Class I arches, the use of a lingual plate can improve denture stability and enhance stress distribution (47). Of course, the self-cleaning properties, and comfort of the lingual bar should also be considered when designing mandibular major connectors (3).

### 3.5 Materials for denture frameworks and clasps

For Kennedy Class I and II, the elastic modulus of varied RPD framework and clasp materials may differentially affect the vertical displacement of distal extension cantilevers, as well as the stress response and elastic deformation of the periodontal ligament and mucosa. Cobalt-chromium (CoCr) clasps exhibit the highest removal force, stiffness, and overall stability, but also generate the greatest stress on abutment teeth (41). In a comparative study, Rodrigues et al. (48) found that commercially pure titanium clasps exerted lower retentive force than CoCr clasps of the same design.

**Figure 4:**
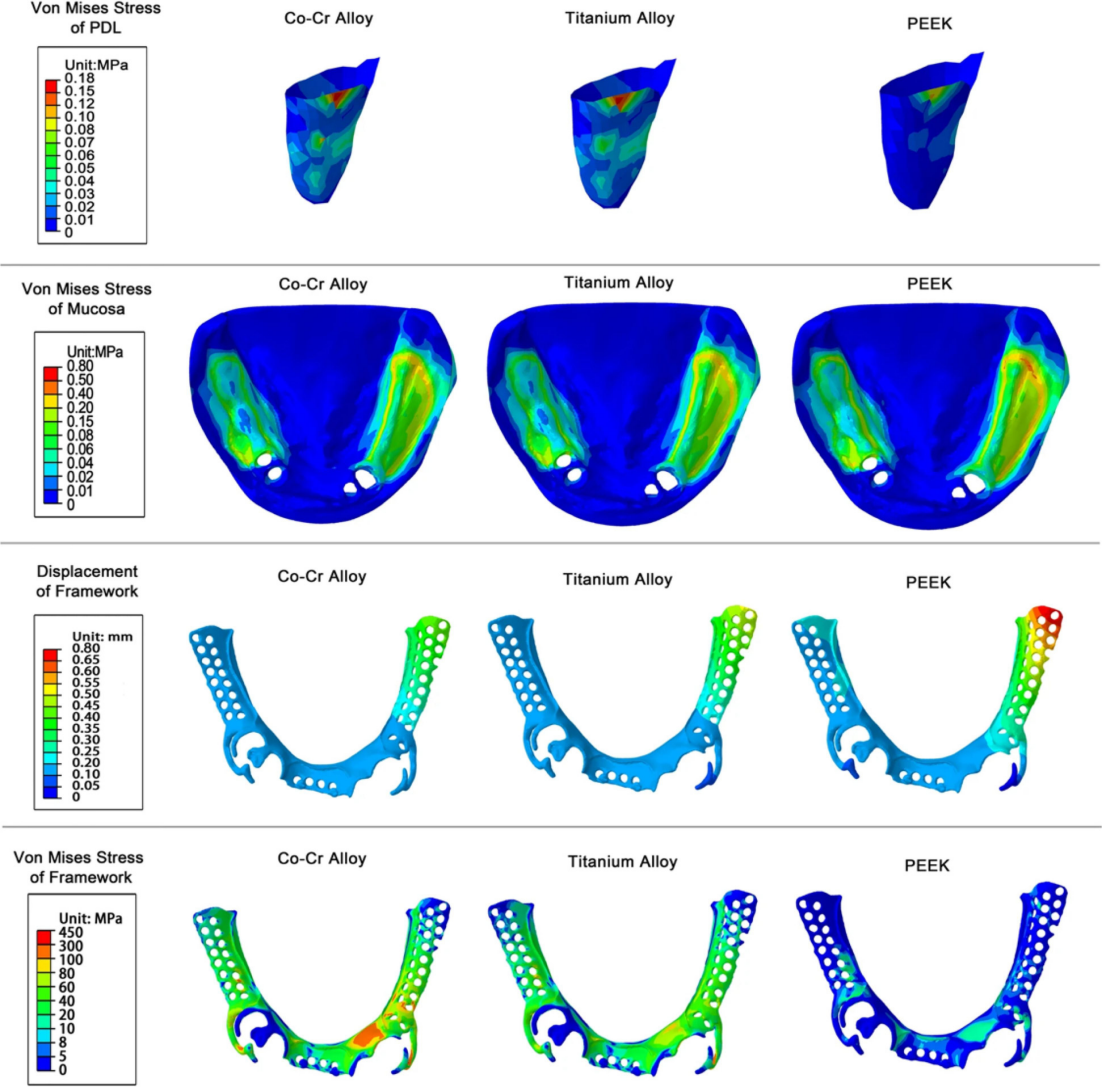
von Mises stress of PDL, mucosa, framework and displacement of framework under vertical loading. Reproduced from (44) under the terms of CC BY 4.0.

Compared to CoCr and titanium alloy (Ti-6Al-4V), polyetheretherketone (PEEK) frameworks and clasps offer greater flexibility. FEA has shown that PEEK frameworks generate the lowest von Mises stress on the periodontal ligament of abutment teeth, which makes PEEK a promising material, particularly for patients with compromised periodontal conditions (44) (Figure 4). While its retentive force is lower than that of CoCr clasps, it is still clinically acceptable, positioning PEEK as a viable alternative for future framework and clasp design (31).

Due to the high rigidity of CoCr alloys, the retentive force generated at the same undercut depth is considerably greater than that of flexible materials such as PEEK (49). To further illustrate the biomechanical differences among conventional and polymer-based clasp materials, the comparative properties of CoCr, PEEK, Polyetherketoneketone (PEKK), and carbon fiber reinforced PEEK (CFR-PEEK) are summarized in Table 3. Luo et al. (51), in an FEA study, reported that the stress concentration in the retentive arm of PEEK Aker clasps was mainly located at the shoulder region. This phenomenon may not be observed in metal clasps, as their higher stiffness means that frictional interaction with the abutment does not substantially affect the metal clasp arm. Therefore, reinforcement of the shoulder region in PEEK clasps is necessary to minimize the risk of fracture.

**Table 3:** Comparative biomechanical properties of coCr, PEEK, PEKK, and CFR-PEEK clasp materials, including retentive force, elastic modulus, and fatigue performance. Data adapted from Gentz et al. (2022) and Bonnheim et al. (2019).

Study (year)	Material	Retentive force (*N*)	Elastic modulus (GPa)	Fatigue/Cycles
Gentz et al. (2022) (49)	CoCr (cast)	∼11.98	∼200–220	Stable through 15,000 cycles (≈10 years simulated use).
Gentz et al. (2022) (49)	PEEK	∼2.16	∼3–4	15,000 cycles: borderline significant retention drop (*p* = 0.039), but ≥ baseline.
Gentz et al. (2022) (49)	PEKK	∼2.74	∼5–6	15,000 cycles: no significant difference with baseline.
Bonnheim et al. (2019) (50)	CFR-PEEK	NA	∼17	Under cyclic loading, CFR- PEEK exhibited an improved resistance to fatigue crack propagation compared with PEEK.

NA, not available.

From a clinical perspective, PEEK frameworks are particularly advantageous for patients with reduced periodontal support, where lowering stress on the abutment teeth is critical for prolonging their prognosis. In addition, for patients with metal allergies or aesthetic concerns about the appearance of metal clasps, tooth-colored PEEK clasps offer a biocompatible and esthetically favorable alternative, although both materials demonstrate comparable retention and patient satisfaction (52). However, the lower elastic modulus of traditional PEEK frameworks also leads to greater cantilever displacement at the distal-extension site, potentially compromising prosthesis stability and reducing masticatory efficiency (44). In cases requiring higher masticatory efficiency or involving long distal-extension bases, clinicians should be cautious of excessive cantilever displacement that may result from the relatively low elastic modulus of PEEK. Beyond conventional PEEK, a number of modifications and novel polymers have been investigated to overcome its mechanical limitations. PEKK replaces one of the flexible ether linkages in PEEK with a more rigid ketone group, resulting in higher mechanical strength, tunable crystallinity, and a broader processing window (53). These properties make it particularly suitable for digital manufacturing and the design of personalized prosthetic frameworks (54). In an FEA study of the All-on-Four full-arch rehabilitation system, PEKK was evaluated as a framework material and, due to its improved ability to dissipate mechanical loads, exhibited lower stress accumulation at the prosthetic screw and denture base interface (55). Clinically, this may correspond to a reduced risk of acrylic base fracture and screw loosening.

Another effective strategy to enhance the mechanical performance of PEEK is the incorporation of inorganic fillers, such as carbon fibers, hydroxyapatite (HAp) (56). CFR-PEEK has an elastic modulus close to the human cortical bone, making it a promising candidate to replace metallic materials (57). In an FEA of mandibular complete-arch implant-supported rehabilitation, the CFR-PEEK framework demonstrated better stress distribution on the implants and surrounding tissues compared to PEEK (58). Specifically, CFR-PEEK effectively reduced cortical bone stress around the distal implants, indicating its potential to enhance biomechanical performance in load-bearing regions. In addition, various reinforcement strategies, such as glass fiber reinforcement (59), zirconia nanofiller reinforcement (60), and HAp nanoparticle reinforcement (61) of PEEK composites, not only improve the processability and mechanical properties of PEEK but also endow it with superior biomechanical performance, highlighting its potential as a high-performance material for future denture frameworks and even dental implants. Collectively, these advances indicate that polymer-based and nanofiller-reinforced materials may represent a paradigm shift in RPD framework design, providing lightweight yet biomechanically favorable alternatives to traditional metals.

## 4 Biomechanical considerations in the design of implant-supported RPDs

Implant-supported RPDs (ISRPDs) are a type of prosthetic rehabilitation in which attachments anchor the denture to implants, with the implant–attachment system providing retention, stability, and support for the prosthesis (62). For Kennedy Class I and II edentulous arches, placing implants in the distal extension area of the edentulous space—particularly in the mandible—can effectively convert a Class I or II arch into a Class III (63). This reduces the stress borne by natural supporting structures and mitigates the biomechanical risks associated with conventional RPDs (64). Especially for patients with multiple missing teeth in distal extension areas, this approach offers a cost-effective and less invasive alternative compared with implant-supported fixed prostheses, and has been recommended as a viable option to harmonize the incompatible resiliency between abutment teeth and distal extension edentulous ridges (65). However, the load-bearing capacity of implants is inferior to that of healthy natural teeth. Excessive occlusal loading on implants may lead to peri-implant bone resorption as well as biological and mechanical complications, such as fracture of the implant or abutment (66). Factors such as implant site selection, length and diameter, and macro- and micro-topography directly influence the biomechanics of implants, and these considerations are equally critical for the long-term prognosis of the prosthesis (67).

### 4.1 Implant site selection

**Figure 5:**
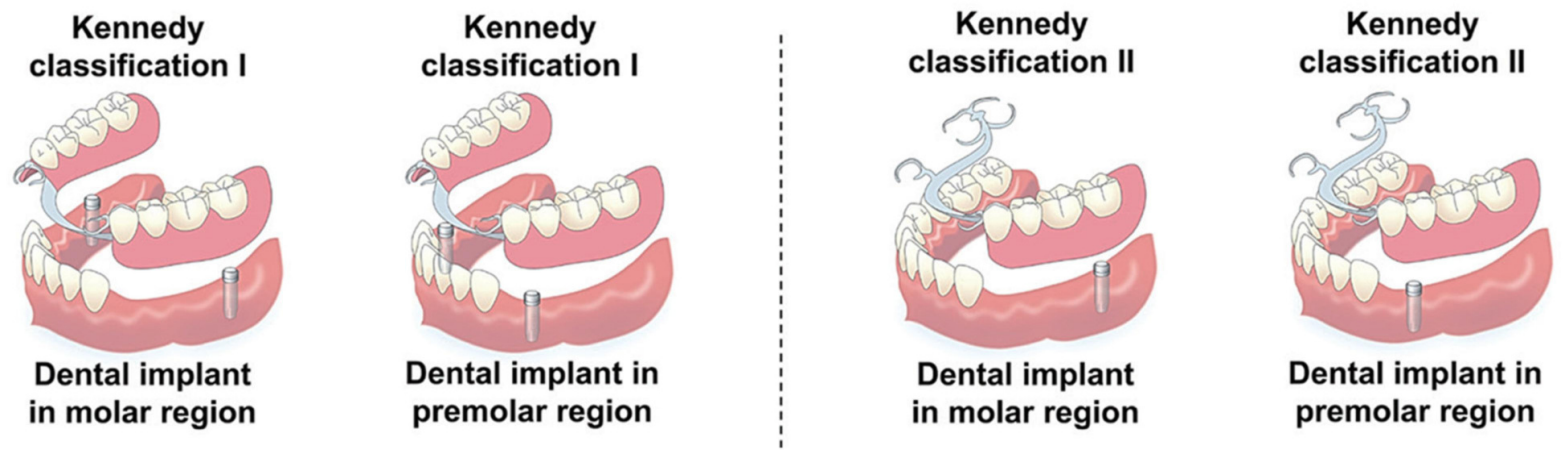
Schematic illustration of implant-supported removable partial dentures (ISRPDs) with different implant site selections for Kennedy Class I and Class II distal-extension cases. Reproduced from (68) under the terms of CC BY 4.0.

For patients with distal-extension posterior edentulism, there is no universally accepted standard for single implant site selection (68) (Figure 5). Theoretical models suggest that placing the implant more distally, at the first or second molar region, can minimize stress on the soft tissue and alveolar bone, whereas placing the implant immediately distal to the terminal abutment tooth may reduce stress on the abutment itself (62). Memari et al. (69) reported in an FEA that implant placement in the second premolar region resulted in the highest stress levels on the implant, abutment, and cancellous bone, while placement in the first molar region produced the lowest stress. Using piezoelectric sensors, Matsudate et al. (70) measured the effect of implant placement at the second premolar (mesial implant) and second molar (distal implant) regions on abutment stress, and found that distal implants subjected the terminal abutment and implant to greater loads, while the residual ridge experienced the least loading.

However, conflicting results have been reported depending on the research methodology. In an edentulous mandibular acrylic resin model, placement of the implant in the second molar region significantly reduced denture base displacement, mesiodistal movement, and abutment bending moment compared to placement in the first molar region (71). Similarly, Hegazy et al. (72) demonstrated in a simulated Kennedy Class I model that implant placement in the second molar region significantly decreased stresses on both the implant and the abutment compared with placement in the first premolar region. Clinical studies have further shown that patients with implants placed in the molar region of implant-supported RPDs reported higher visual analogue scale (VAS) scores than those with premolar implants, with 56.7% of subjects preferring molar implant support. Nevertheless, no significant differences were observed between molar and premolar sites in terms of the Mixing Ability Index (MAI) or radiographic implant parameters (73).

### 4.2 Implant length and diameter

The length and diameter of implants influence the displacement and stress distribution of abutment teeth, implants, and the surrounding bone. Fayaz A et al. (74) demonstrated through finite element analysis that increasing implant length can reduce stress on the terminal abutment and minimize stress on the surrounding bone—particularly cancellous bone—although it increases the stress borne by the implant itself. Another study reported that increasing implant length significantly decreased denture displacement and von Mises stress values on the implant, whereas increasing implant diameter had a significant effect on reducing von Mises stress but did not influence denture displacement (75). Similarly, finite element analysis has also shown that wider implants reduce von Mises stress in the cancellous bone at the apical region of terminal abutments (76).

### 4.3 Implant angulation

Fayaz et al. (74) evaluated the effect of different implant angulations on stresses in abutment teeth and implants, and found that as the angulation increased, implant stress gradually rose and concentrated at the implant neck, while stress concentration in the periodontal ligament of the terminal abutment decreased. A finite element analysis further demonstrated that applying a mesial angulation ranging from 5° to 30° to implants placed in the second molar region reduced vertical displacement of the mucosa in mandibular distal-extension implant-supported RPD models (77). Compared with vertically positioned implants, slightly inclined implants (approximately 5°) provided more favorable stress distribution. However, the biomechanical mechanisms and long-term effects of varying implant angulations remain insufficiently studied, and no reports have been found on the influence of implant angulation in the maxilla, which may be due to the fact that maxillary implant positioning is more constrained by the size and location of the maxillary sinus rather than biomechanical considerations.

### 4.4 Macro- and micro-geometry of implants

The macro-shape of implants (cylindrical vs. conical) and their micro-surface features (thread shape, pitch, and thread depth) influence the interfacial stress distribution at the bone–implant surface (78–80). Finite element analysis studies have shown that conical implants exhibit higher peak von Mises stress values than cylindrical implants across all bone types, possibly due to stress concentration occurring at the sharp lateral angles of conical designs. In Class I and II bone, cylindrical implants demonstrate higher success rates than conical implants, likely because they generate less lateral force within these bone qualities. In all models, the distal region of the implant consistently recorded the highest von Mises stress, and increasing thread depth was associated with a reduction in von Mises stress (81). From a biomechanical perspective, cylindrical implants are therefore recommended to minimize lateral stress on cancellous bone. Moreover, in situations involving high occlusal forces, short implant lengths, or low bone density, reducing thread pitch and increasing thread depth can maximize the bone–implant contact area and improve primary stability (79).

## 5 Toward improved patient satisfaction: the role of FEA in RPD design and clinical considerations

### 5.1 Causes of patient dissatisfaction with RPDs: insights from FEA

Studies have shown that patient satisfaction with mastication in RPDs is closely associated with oral health-related quality of life (OHRQoL) (82). RPDs with Co-Cr frameworks can achieve greater occlusal force, which is generally perceived as more satisfactory by patients (83). However, their high rigidity also leads to stress concentration in the periodontal ligament of abutment teeth and at clasp–tooth contact areas, potentially causing discomfort or compromising abutment prognosis (84). High-performance polymers such as PEEK demonstrate lower periodontal stress in finite element analyses, but their relatively low elastic modulus results in greater distal cantilever displacement, which clinically manifests as reduced masticatory efficiency and looseness of the prosthesis (44). Such insufficiency in retention and stability is a common cause of patient dissatisfaction (25). In contrast, ISRPDs can effectively improve retention and stability, and thus generally yield higher patient satisfaction compared with conventional RPDs (85). In distal-extension cases, although clinical experience may help avoid overtly unfavorable designs, it cannot quantify stress distribution on abutments, denture bases, and mucosa, nor provide sufficient reference for new materials such as PEEK and PEKK. Consequently, issues such as saddle displacement, denture instability, reduced masticatory efficiency, and mucosal pain may not be fully prevented. If multiple loading conditions are considered and different design options compared during the pre-design stage, FEA can still serve as a risk-warning tool, assisting clinicians in identifying and avoiding unfavorable biomechanical patterns at the outset, thereby improving patient satisfaction.

Of course, patient satisfaction with RPDs is not determined solely by biomechanical factors. The exposure of metal clasps in the anterior region often leads to esthetic dissatisfaction, whereas PEEK clasps, due to their tooth-colored appearance, are generally more acceptable esthetically (86). Wearing comfort is also critical; although extended denture bases can enhance support and reduce mucosal soreness, excessive extension may trigger the gag reflex and lower satisfaction (87, 88). Individual factors such as saliva viscosity, mucosal thickness and viscoelasticity and gag reflex are rarely incorporated into FEA models. Most FEA simulations assume homogeneous, linear material properties under static loading, whereas clinical masticatory forces are dynamic, cyclic, and multidirectional. Furthermore, many numerical models oversimplify the complex biomechanical behavior of oral mucosa, simulating only static or elastic responses and neglecting dynamic and viscoelastic characteristics (89). As a result, discrepancies often arise between computational predictions and patients' actual experiences, leading to distortion in FEA outcomes. These limitations collectively explain why, even for prostheses that perform well in FEA analyses, patients may still report discomfort, esthetic concerns, food impaction, or difficulty in adaptation.

### 5.2 FEA-guided strategies for better patient acceptance

Although FEA cannot account for all clinical variables, it remains an important tool for optimizing RPD design and reducing patient dissatisfaction. FEA-based denture optimization enables efficient, patient-specific design and provides quantitative guidance for adjustments, such as balancing clasp rigidity with retention and major connector stiffness with perceived bulkiness. From the patient's perspective, optimized dentures can reduce discomfort and alveolar ridge resorption, thereby extending the interval before further adjustments are required (90).

At the material level, the application of high-performance polymers guided by FEA represents another key direction. Studies have shown that novel materials such as PEKK and CFR-PEEK exhibit more favorable stress distribution (55, 58), yet their properties remain relatively unfamiliar to dental technicians. Incorporating their mechanical parameters into FEA can further optimize their use in RPD fabrication, enabling a better balance among patient-specific anatomical conditions, esthetic and comfort requirements, and the mechanical performance of the prosthesis, which is expected to improve patient satisfaction. Ultimately, FEA serves as a bridge between biomechanical optimization and clinical practice, providing a rational basis for prosthetic design improvements and achieving dual gains in functional performance and patient acceptance.

## 6 Construction of patient-specific biomechanical models and personalized FEA-based analysis

Although numerous design concepts for RPDs have been proposed based on FEA, photoelastic stress analysis, and clinical experience, their practical clinical application remains significantly limited. A key challenge lies in the substantial inter-individual variability in oral anatomical structures, physiological function, and biomechanical environments, which makes standardized designs suboptimal. If correlations between alveolar ridge morphology and denture movement can be established through FEA, practical and objective criteria for personalized design may be developed (91).

Recent studies have combined digital technologies with individualized modeling approaches. Using 3D scanning and computed tomography (CT) data, researchers can reconstruct patient-specific oral structures and apply FEA to evaluate stress distribution (92–94). This allows for targeted biomechanical optimization of RPD components, improving stability, comfort, and overall performance. CAD and CAD/CAM-based digital frameworks have already shown promising outcomes in terms of retention, functional adaptation, and patient satisfaction (15). However, intelligent RPD design platforms that integrate FEA remain scarce. Kibi et al. (95) developed a CAD-based RPD system with embedded FEA functionality. Digital impressions were obtained using 3D scanning to generate mucosal models, artificial teeth were positioned using a virtual occlusal plane, and FEA modules were used to analyze mucosal stress distribution under various loading conditions—guiding the placement of functional cusps for improved biomechanical performance.

## 7 Next-generation RPD design: AI-enhanced FEA modeling

In recent years, artificial intelligence (AI)—particularly machine learning and deep learning algorithms—has demonstrated strong capabilities in medical image analysis, 3D morphology recognition, and complex data prediction (96–98). AI is gradually being integrated into several key stages of the digital workflow for removable partial dentures (RPDs), including: 1) Detection and classification of partial edentulism: Convolutional neural networks (CNNs) can automatically analyze intraoral photographs, radiographs, or intraoral scans to identify missing teeth (1, 99), determine the edentulous pattern, and classify cases based on systems such as the Kennedy classification (99), thereby improving diagnostic efficiency and supporting rapid treatment planning. 2) Intelligent framework design recommendation: Decision-making models based on deep learning or knowledge graphs can recommend appropriate connectors, clasp types, and spatial arrangements according to specific clinical conditions, enabling semi- or fully automated CAD design of RPD frameworks (100, 101). 3) 3D denture model generation: Generative adversarial networks (GANs) and other models have been used to reconstruct 3D framework geometry automatically, shifting the design process from a traditional linear pipeline to a data-driven structural optimization workflow (95). 4) Expert system and rule-based training: By integrating large case libraries and clinical expertise, AI-powered expert systems can simulate the decision-making patterns of experienced clinicians. Rule-based learning further enhances interpretability and clinical control of AI-generated outputs (99).

### 7.1 Emerging pathways for AI-driven biomechanical prediction

Current AI technologies still face challenges in addressing one of the most critical issues in distal-extension RPD design—stress concentration and its potential damage to abutments, mucosa, and distal extension tissues. Effectively mitigating such biomechanical risks requires accurate quantification of stress distribution in these key tissue areas (1). In addition, substantial inter-individual variation in mandibular bone morphology, bone density, and occlusal behavior must be considered when designing patient-specific biomechanical responses (102). Embedding finite element analysis (FEA) within AI-assisted RPD design frameworks offers a promising path toward truly personalized, biomechanically optimized prosthetic strategies. Such systems would integrate real patient anatomy and loading conditions to drive intelligent design.

Emerging studies have explored AI-driven biomechanical prediction models as alternatives to conventional FEA simulations (103, 104). For instance, supervised neural networks have been trained to rapidly predict distributions of maximum principal or shear stress in key areas based on denture geometry and load location (105, 106). These approaches maintain accuracy while dramatically improving computational efficiency and automation.

AI and FEA are inherently complementary in RPD design. On one hand, AI can be trained on large volumes of FEA-generated data to rapidly infer the relationship between design parameters and stress outcomes, thereby reducing simulation cycles. On the other hand, AI algorithms can extract fine-grained features—such as supporting tissue morphology, individual mucosal elasticity ranges, and occlusal contact points—from intraoral scans, providing more precise and personalized input conditions for FEA models (107, 108).

### 7.2 Key challenges for AI–FEA integration

In the long term, to truly realize a closed-loop optimization system driven by AI and informed by FEA feedback, three major challenges must be addressed: algorithm architecture, data infrastructure, and system efficiency. At the algorithmic level, it is necessary to develop optimization methods that support multi-objective search and structural adaptivity—such as genetic algorithms, Bayesian optimization, and reinforcement learning—which have already shown promise in dental implant design via FEA optimization (109, 110). Likewise, the exploration of graph neural networks (GNNs) in modeling dental arch topology and force-transmission pathways is supported by recent advances in GNN-based surrogate FEA frameworks (111). From a data perspective, the absence of large-scale, integrated databases combining RPD design, simulation, and clinical feedback remains a major bottleneck that limits generalizability and reliability. In addition, the high computational cost of automated FEA continues to pose a challenge. Deep learning–based surrogate models have demonstrated substantial value in related engineering applications, where they reduced simulation time by over 100-fold while preserving accuracy, with errors typically within 5% (112, 113).

Future research should particularly focus on these aspects:

1. Validation and Clinical Translation: At present, most AI–FEA studies remain confined to virtual simulations, with limited validation against real patient outcomes. To enhance translational value, future investigations should incorporate prospective clinical trials, patient-reported outcomes (such as comfort, retention, and masticatory efficiency), and long-term prosthesis survival data across diverse populations (114, 115).
2. Data Limitations and Bias: In addition to the overall scarcity of data, issues of imbalance and bias are evident, including the underrepresentation of certain Kennedy classifications, specific age groups, and anatomical variations. Overcoming these challenges will require the establishment of multicenter collaborative datasets and the implementation of federated learning approaches, which enable large-scale training while ensuring data privacy and fairness (116, 117).
3. Integration with Clinical Workflow: Although the complementary strengths of AI and FEA are increasingly recognized, their integration into routine prosthodontic workflows remains limited. A feasible implementation pathway could involve intraoral scanning to capture three-dimensional anatomical structures, AI-based preprocessing and preliminary design, real-time biomechanical optimization through embedded FEA modules, and subsequent transfer of the finalized design to digital manufacturing systems. Such a streamlined process would facilitate translation from theoretical modeling to practical clinical application.
4. Ethical and Regulatory Considerations: The rapid adoption of AI in dentistry raises important ethical and legal challenges, particularly concerning patient data security, algorithm transparency, and liability in the event of treatment failure. Addressing these issues requires the development of regulatory standards, mechanisms for informed patient consent, and clear guidelines for accountability, all of which are essential to ensure the safe and responsible deployment of AI-driven denture design (114, 118).

In summary, intelligent RPD design platforms that integrate multimodal data modeling, interpretable AI techniques, and real-time biomechanical feedback hold great potential to transcend the limitations of traditional CAD systems and provide truly intelligent, personalized solutions for prosthodontic rehabilitation.

## 8 Conclusion

In summary, FEA provides an indispensable biomechanical perspective for the rational design of distal extension RPDs, particularly by clarifying stress transmission in abutments, mucosa, and distal saddle areas. The novelty of this study lies in highlighting how FEA can not only identify biomechanical risks but also serve as a design optimization tool when integrated with digital workflows and emerging AI technologies. By focusing on Kennedy Class I and II scenarios, this review underscores the importance of balancing stress distribution, retention, and patient satisfaction. Ultimately, FEA bridges biomechanical theory and clinical outcomes, paving the way for next-generation, intelligent, patient-specific RPD rehabilitation.
